# Improving the TIR3B oncological stratification: try to bridge the gap through a comprehensive presurgical algorithm

**DOI:** 10.1007/s40618-023-02182-5

**Published:** 2023-09-22

**Authors:** C. Sparano, M. Puccioni, V. Adornato, E. Zago, B. Fibbi, B. Badii, L. Bencini, G. Mannelli, V. Vezzosi, M. Maggi, L. Petrone

**Affiliations:** 1 Endocrinology Unit, Department of Experimental and Clinical Biomedical Sciences ‘Mario Serio’, University of Florence, Florence, Italy; 2 Endocrinology Unit, Medical-Geriatric Department, Careggi University Hospital, Viale Pieraccini 18, 50139 Florence, Italy; 3 Unit of Endocrine Surgery, Careggi University Hospital, Florence, Italy; 4 Division of General Surgery, Department of Oncology and Robotic Surgery, Careggi University Hospital, Florence, Italy; 5 Head and Neck Oncology and Robotic Surgery, Department of Experimental and Clinical Medicine, University of Florence, 50134 Florence, Italy; 6 Department of Histopathology and Molecular Diagnostics, Careggi University Hospital, Florence, Italy; 7 Consorzio I.N.B.B, 00136 Rome, Italy

**Keywords:** Indeterminate cytology, Thyroid cancer, Cytology, Algorithms

## Abstract

**Purpose:**

Indeterminate cytology still puzzles clinicians, due to its wide range of oncological risks. According to the Italian SIAPEC–IAP classification, TIR3B cytology holds up to 30% of thyroid cancer, which justifies the surgical indication, even if more than half of cases do not result in a positive histology. The study aim is to identify potential clinical, ultrasound or cytological features able to improve the surgical indication.

**Methods:**

Retrospective analysis. A consecutive series of TIR3B nodules referred to the Endocrine Unit of Careggi Hospital from 1st May 2014 to 31st December 2021 was considered for the exploratory analysis (Phase 1). Thereafter, a smaller confirmatory sample of consecutive TIR3B diagnosed and referred to surgery from 1st January 2022 to 31st June 2022 was considered to verify the algorithm (Phase 2). The main clinical, ultrasound and cytological features have been collected. A comprehensive stepwise logistic regression was applied to build a prediction algorithm. The histological results represented the final outcome.

**Results:**

Of 599 TIR3B nodules referred to surgery, 451 cases were included in the exploratory analysis. A final score > 14.5 corresponded to an OR = 4.98 (95% CI 3.24–7.65, *p* < 0.0001) and showed a PPV and NPV of 57% and 79%, respectively. The Phase 2 analysis on a confirmatory sample of 58 TIR3B cytology confirmed that a threshold of 14.5 points has a comparable PPV and NPV of 53% and 80%, respectively.

**Conclusions:**

A predictive algorithm which considers the main clinical, US and cytological features can significantly improve the oncological stratification of TIR3B cytology.

**Supplementary Information:**

The online version contains supplementary material available at 10.1007/s40618-023-02182-5.

## Introduction

Indeterminate thyroid cytology is still considered the clinical *graveyard* of the thyroidologist, due to the wide range of oncological risks provided by its results. In 2014, the Italian Society for Anatomic Pathology and Cytology joined with the Italian Division of the International Academy of Pathology (SIAPEC–IAP) [1] tried to refine this category by splitting indeterminate cytology into two subgroups, i.e., TIR3A and TIR3B. The former—also labelled as a low-risk indeterminate lesion (LRIL)—is characterized by a high cellularity, possible degenerative aspects, and various proportion of microfollicular structures, but inadequate to define a follicular neoplasm. This cytology is comparable to the “atypia of undetermined significance or follicular lesion” (AUS/FLUS) of the USA Bethesda classification [2] and discloses an estimated malignancy risk of < 10%. Therefore, a cytological rechallenge, followed by clinical monitoring, is currently suggested. On the other side, TIR3B cytology—also called a high-risk indeterminate lesion (HRIL)—discloses a higher and redundant cellularity with microfollicular configuration and/or prevalent Hürthle cells (HC). The corresponding Bethesda cytological category is “follicular neoplasm or suspicious for a follicular neoplasm” (FNs) [2], and the expected malignancy risk, ranges from 15% to 30% [1]. Accordingly, a surgical indication is always recommended [1], more for a diagnostic, rather than a curative purpose.

However, the present guidelines argue that the aforementioned malignancy rates are supported by limited evidence, even if comparable to other international classifications. Therefore, large and reliable studies are required to understand the real oncological risk of these cytological categories [1].

Several attempts have been made to improve surgical indications, usually with disappointing results. For instance, thyroid nodules ultrasound (US) scores were less effective in the malignancy prediction of the indeterminate cytology [3]. Of note, most of the available studies excluded this category, due to its worse impact on the overall scores [3–5] and the few promising data came from small cohort samples, requiring further insights to conclude their value [3, 6–10]. Besides, the available thyroid nodule scores have been built mostly to diagnosis papillary thyroid cancer [11]. Conversely, indeterminate cytology disclose more often follicular variant of papillary thyroid carcinoma [12] or other less frequent histology (i.e., follicular thyroid carcinoma or Hürthle cells carcinoma), which reduce the performance of these tools.

The molecular pre-surgical approaches on various cohorts of indeterminate cytology, including TIR3A and TIR3B samples, showed promising results, but at high costs, which are less affordable for a routine outpatient use [13, 14].

A meta-analysis, considering several cytological classifications, showed that fluorine-18-fluorodeoxyglucose positron emission tomography/computed tomography (^18^F-FDG PET/CT) has a marginal ability in selecting indeterminate nodules with a forthcoming positive histology [15]. Once again, in our opinion, ^18^F-FDG PET/CT outcomes do not balance the cost of systematic use of this second-level diagnostic, which should still be reserved for very selected cases.

Finally, several years after the Italian classification was released, growing evidence about higher rates of malignancy for TIR3B cytology is available [16–19], prompting a critical reappraisal of the current estimates [1].

Based on these premises, the present study aims to analyse, in a large and consecutive series of TIR3B nodules systematically referred to surgery, the potential clinical, US, and cytological features able to better predict an oncological outcome. We, therefore, conducted a two-phase analysis. The first phase (exploratory analysis) aimed to construct a reliable algorithm, based on significant clinical and cytological futures, that could predict an unfavourable outcome in a large sample of TIR3B nodules. The second phase (confirmatory analysis) aimed to verify the validity of the constructed algorithm in an independent, small sample of TIR3B nodules. We specifically focused on the role of secondary cytological features that accompany the detailed report of these patients and the prediction values of a comprehensive risk assessment.

## Methods

### Exploratory sample (phase 1)

A consecutive series of 6586 cytology tests performed at the Endocrine Unit of Careggi Hospital between 1st May 2014 and 31st December 2021 has been considered for the exploratory sample. Inclusion criteria: (i) TIR3B cytology (*n* = 599); (ii) accepted surgical indication; (iii) availability of clinical, US, cytological, and histological reports. Exclusion criteria: (i) cytological investigations and histology performed outside of Careggi Hospital; (ii) denial of surgery.

The main clinical information [age, gender, and diagnosis of chronic autoimmune thyroiditis (CAT)] has been collected for each patient. Thyroid US examinations and cytology diagnostics have been performed by an experienced team of five endocrinologists, with specific skills in thyroid disease management. Of note, four physicians were responsible for the US descriptions: they underwent specific training and the consistency of their examination has been verified in a previous study [3], showing a substantial agreement at the Cohen’s *κ* (up to 0.73).

After having gained specific informed consent, each patient underwent a full neck US examination, which has been systematically recorded in a defined outpatient form, including a complete nodule description, according to a standardized lexicon [20, 21] (Supplementary Table 1). Each fine-needle aspiration (FNA) has been performed under US guidance with the capillarity technique with a 21–23 Gauge needle. The FNA sample was immediately rinsed into CytoLyt Solutions (CyticMalborough, MA, USA), and centrifuged to be processed by ThinPrep^®^Processor under PreservCyt Solution. Thereafter thin-layer slides were obtained by the Papanicolaou procedure. Each cytology has been classified according to SIAPEC–IAP classification [1], by two expert pathologists (V.V. and S.B.) with specific training in thyroid pathology. For each cytological report, a series of secondary cytological features have been collected, i.e., the presence/absence of cellular atypia, colloid, aggregate disposition (i.e., monomorphic and redundant cells aggregate), macrophages, plasmacytoid, nuclear pseudo inclusion, HC, anisonucleosis and the kind of cellular configuration (none, follicular, papillary, mixed).

All the histology has been classified, according to the AJCC 2017 [22].

Considering the potential role of autoimmune disease on the cytological results, the presence of CAT was collected for all the included cases, by clinical screening (i.e., a previous diagnosis of thyroiditis with the presence of thyroid auto-antibodies) and verified on histological samples after surgery.

### Confirmatory sample (phase 2)

Clinical, US and cytological risk factors included in a specific algorithm able to detect thyroid malignancy, as derived from the Phase 1 exploratory sample, were retested in an independent and consecutive second cohort of TIR3B nodules (Phase 2 confirmatory analysis) collected between January 1st 2022, and June 30th 2022. Among 378 cytological results, a TIR3B result was present in 58 nodules and referred to surgery. The same Phase 1’s inclusion and exclusion criteria have been applied to this new population.

The Local Ethics Committee (Comitato Etico Area Vasta Centro—CEAVC, Florence, Tuscany, Italy) approved the study and it was conducted in compliance with the Declaration of Helsinki principles.

### Statistical analysis

Continuous variables have been expressed as the mean ± standard deviation when normally distributed or median [interquartile range] when non-normally distributed. Categorical variables have been expressed as numbers and percentages. T-student or Mann–Whitney tests have been applied to assess differences in normally or non-normally distributed continuous variables, respectively. Chi-square tests have been used to compare categorical variables. A binomial test explored eventual differences between the rates of thyroid cancer in the present population, compared to the guidelines [1]. Receiver Operating Characteristic (ROC) curve analysis was applied to find the best cut-offs for continuous variables (i.e., age and nodules size) and to analyse the accuracy. The histological result (positive/negative for malignancy) has been used as the outcome. According to the Akaike Information Criterion (AIC), a stepwise logistic regression has been performed to find the best prediction model, using the histological outcome (a positive result) as the readout. Considering the significant variables of the prediction model, we built up a predictive algorithm, based on the weight of each odd ratio (OR). Positive predictive value (PPV) and negative predictive value (NPV) have been calculated. The analyses have been performed with SPSS version 28.0, R software [23], and Jamovi software [24].

## Results

### Exploratory sample (phase 1)

A total of 599 TIR3B cytology results (9.1% of all cytological results) have been considered for the training analysis. Of those, 148 have been excluded for the absence of histology (*N* = 80) or fundamental information (*N* = 68), as specified in the exclusion criteria, i.e., cytological investigation and histology performed outside our hospital. A final cohort of 451 subjects was eligible for the study and was included in the final exploratory modelling. Considering the expected rate of positive histology (< 30%) [1], a significantly higher tumour rate has been observed in real practice (36%), *p* = 0.010 (Table 1). Table 2 shows the histological results of surgery, while Table 3 shows a descriptive analysis of the overall sample and its stratification according to the historical outcome after surgery. Briefly, the most frequent histological variant was the follicular variant of papillary thyroid cancers and patients with a positive histology showed several differences in clinical, US, and cytological features. From a clinical perspective, the cohort with a positive histology showed a younger age (*p* < 0.001) and a higher rate of CAT (*p* = 0.024) than the rest of the sample. Considering nodule features, a lower nodule size (15 vs. 21 mm, *p* < 0.001), a hypoechoic aspect at US (*p* < 0.001), a solid composition (*p* = 0.018), and a higher rate of the mixed vascular pattern (*p* = 0.021) characterized patients with a positive histology. Considering cytological features, fewer presence of colloid (*p* = 0.016), mostly follicular patterns (*p* < 0.001), lower presence of HC (*p* = 0.002), higher anisonucleosis rates (*p* < 0.001) and mostly aggregate disposition (*p* < 0.001) have been observed in patients with a positive histology. The simultaneous presence of HC and CAT was found in 36 patients (8.0%), without significant differences in the histological outcome (*p* = 0.305).

**Table 1:** Binomial test according to the maximum expected rate of positive histology

Level	Count	Total	Proportion	*p*
Histology				
Negative	290	451	0.643	**< .001**
Positive	161	451	0.357	**0.010**

Bold numbers highlight significant differences

H_a_ is proportion ≠ 0.3

**Table 2:** Histological variant of positive and negative histology according to the exploratory or confirmatory samples

Histological variant of thyroid cancer (TC)	Exploratory sample *n* = 451	Confirmatory sample *n* = 58
**Positive histology**		
Follicular variant of papillary TC *n* (%)	63 (14.0)	6 (10.3)
Papillary classic TC *n* (%)	38 (8.4)	5 (8.6)
Minimally invasive follicular TC *n* (%)	19 (4.2)	–
Hürthle cell TC *n* (%)	13 (2.9)	1 (1.7)
Oncocytic variant of TC *n* (%)	13 (2.9)	1 (1.7)
Solid variant of TC *n* (%)	6 (1.3)	3 (5.2)
Insular variant of TC *n* (%)	5 (1.1)	1 (1.7)
Follicular TC *n* (%)	2 (0.4)	–
Cribriform TC *n* (%)	1 (0.2)	–
Tall cell variant TC *n* (%)	1 (0.2)	–
**Negative histology**		
Follicular adenoma *n* (%)	131 (29.1)	15 (25.9)
Hürthle cell adenoma *n* (%)	71 (15.7)	11 (19.0)
Nodular goitre *n* (%)	59 (13.1)	7 (12.1)
Adenomatoid hyperplasia *n* (%)	15 (3.4)	3 (5.2)
Neoplasm with papillary-like nuclear features (NIFTP) *n* (%)	12 (2.7)	2 (3.4)
Follicular tumour of uncertain malignant potential (FT-UMP) *n *(%)	2 (0.4)	3 (5.2)

**Table 3:** Overview of the whole training sample and according to the histological outcome

Factor	Overall (*N* = 451)	Histology (*N* = 451)		*p* value
		Negative (*N* = 290)	Positive (*N* = 161)	
Clinical features				
Age				
Years	55.74 (14.30)	57.96 (13.31)	51.75 (15.17)	**< 0.001**
Gender				
Female	342 (75.8)	222 (76.6)	120 (74.5)	0.647
Male	109 (24.2)	68 (23.4)	41 (25.5)	
Chronic autoimmune thyroiditis				
Absent	329 (73.8)	223 (77.4)	106 (67.1)	**0.024**
Present	117 (26.2)	65 (22.6)	52 (32.9)	
Ultrasound features				
Nodule size (mm)	20.80 (10.94)	21.00 [15.00, 28.00]	15.00 [10.00, 23.50]	**< 0.001**
Hypoechogenicity				
Absent	143 (31.8)	114 (39.4)	29 (18.1)	**< 0.001**
Present	306 (68.2)	175 (60.6)	131 (81.9)	
Solid composition				
Absent	50 (11.1)	40 (13.8)	10 (6.2)	**0.018**
Present	401 (88.9)	250 (86.2)	151 (93.8)	
Taller-than-wide shape				
Absent	391 (87.7)	253 (87.8)	138 (87.3)	0.881
Present	55 (12.3)	35 (12.2)	20 (12.7)	
Microcalcification				
Absent	366 (81.2)	238 (82.1)	128 (79.5)	0.531
Present	85 (18.8)	52 (17.9)	33 (20.5)	
Nodule vascular pattern				
I	21 (4.7)	10 (3.5)	11 (7.0)	**0.021**
II	278 (62.2)	194 (67.1)	84 (53.2)	
III	25 (5.6)	13 (4.5)	12 (7.6)	
Mixed	123 (27.5)	72 (24.9)	51 (32.3)	
Cytological features				
Cellular atypia				
Absent	448 (99.3)	289 (99.7)	159 (98.8)	0.291
Present	3 (0.7)	1 (0.3)	2 (1.2)	
Colloid				
Absent	13 (2.9)	4 (1.4)	9 (5.6)	**0.016**
Present	438 (97.1)	286 (98.6)	152 (94.4)	
Configuration				
None	133 (29.5)	101 (34.8)	32 (19.9)	**< 0.001**
Papillary	5 (1.1)	2 (0.7)	3 (1.9)	
Follicular	300 (66.5)	183 (63.1)	117 (72.7)	
Mixed	13 (2.9)	4 (1.4)	9 (5.6)	
Plasmacytoid				
Absent	450 (99.8)	290 (100)	160 (99.4)	0.357
Present	1 (0.2)	0 (0)	1 (0.6)	
Nuclear pseudo inclusion				
Absent	449 (99.6)	289 (99.7)	160 (99.4)	1.000
Present	2 (0.4)	1 (0.3)	1 (0.6)	
Hürthle cells				
Absent	273 (60.5)	160 (55.2)	113 (70.2)	**0.002**
Present	178 (39.5)	130 (44.8)	48 (29.8)	
Anisonucleosis				
Absent	199 (44.1)	149 (51.4)	50 (31.1)	**< 0.001**
Present	252 (55.9)	141 (48.6)	111 (68.9)	
Macrophages				
Absent	205 (45.5)	123 (42.4)	82 (50.9)	0.093
Present	246 (54.5)	167 (57.6)	79 (49.1)	
Aggregate disposition				
Absent	125 (27.7)	99 (34.1)	26 (16.1)	**< 0.001**
Present	326 (72.3)	191 (65.9)	135 (83.9)	

Bold numbers highlight significant differences between negative and positive histology

To find the best thresholds for predicting malignancy for the continuous variables age and nodular size, ROC curve analysis has been performed, using the histological results as a readout. Regarding age, a threshold value of 55 years showed a sensitivity of 58.6% and a specificity of 58.4% (AUC = 0.625, 95% CI 0.57–0.68, *p* < 0.0001) in predicting histological outcome. Similarly, a size cut-off of 18 mm showed a sensitivity and a specificity of 65.9% and 61.2%, respectively (AUC = 0.675, 95% CI 0.619–0.730, *p* < 0.0001).

Considering all the significant categorical variables—as in Table 3—and those derived from the aforementioned ROC analyses (i.e., age ≥ 55 years and size ≥ 18 mm), a stepwise multivariate analysis by the Akaike Information Criterion (AIC) has been performed. Table 4 shows the significant features included in the best-fitting model. Of note, age ≥ 55 years (OR = 0.489), nodule size ≥  18 mm (OR = 0.354) along with the presence of colloid within the cytological report (OR = 0.181) all represent favourable features, at odds with CAT (OR = 1.74), hypoechogenicity (OR = 2.79), HC (OR = 4.2), anisonucleosis (OR = 5.15), aggregate disposition (OR = 4.55), that represented unfavourable features (Table 4). Please note that in the final adjusted model, as in Table 4, HC presence appeared as an unfavourable prognostic factor, at variance with Table 3.

**Table 4:** Stepwise multivariate analysis by AIC, considering the most significant population features and using the histological outcome as readout

	Odd ratio	Confidence interval 95%		*p* value
		Lower	Upper	
Age ≥ 55 years	0.489	0.3100	0.773	**0.0022**
CAT	1.740	1.0500	2.900	**0.0323**
Hypoechoic nodule	2.790	1.6200	4.810	**0.0002**
Nodule size ≥ 18 mm	0.354	0.2250	0.557	**0.0000**
Hürthle cell	4.200	1.2500	14.10	**0.0205**
Anisonucleosis	5.150	1.6400	16.10	**0.0049**
Colloid	0.181	0.0370	0.881	**0.0343**
Aggregate disposition	4.550	1.5700	13.20	**0.0053**
Follicular configuration	0.475	0.1870	1.200	0.1160

Bold numbers highlight significant *p* values

AIC = 474.91

Area under the curve 0.769 95% CI 0.721–0.817

*AIC* Akaike information criterion, *CAT* chronic autoimmune thyroiditis

**Table 5:** Overview of the confirming sample

Factor	Total sample (*N* = 58)
Clinical features	
Age	
Years	53.74 ± 14.03
Gender	
Female	51 (87.9)
Male	7 (12.1)
Chronic autoimmune thyroiditis	
Absent	41 (10.7)
Present	17 (29.3)
Ultrasound features	
Nodule size (mm)	16 [12, 24]
Hypoechogenicity	
Absent	35 (60.3)
Present	23 (39.7)
Solid composition	
Absent	4 (6.9)
Present	54 (93.1)
Taller-than-wide shape	
Absent	57 (98.2)
Present	1 (1.8)
Microcalcification	
Absent	54 (93.1)
Present	4 (6.9)
Nodule vascular pattern	
I	3 (5.2)
II	32 (55.2)
III	0 (0)
Mixed	23 (39.7)
Cytological features	
Cellular atypia	
Absent	58 (100)
Colloid	
Absent	1 (1.7)
Present	57 (98.3)
Configuration	
None	27 (48.6)
Papillary	0 (0)
Follicular	27 (46.6)
Mixed	4 (6.9)
Plasmacytoid	
Absent	58 (100)
Nuclear pseudo inclusion	
Absent	58 (100)
Hürthle cells	
Absent	33 (56.9)
Present	25 (43.1)
Anisonucleosis	
Absent	28 (48.3)
Present	30 (51.7)
Macrophages	
Present	58 (100)
Aggregate disposition	
Absent	28 (48.3)
Present	30 (51.7)
Histology	
Negative	41 (70.7)
Positive	17 (29.3)

**Fig. 1:**
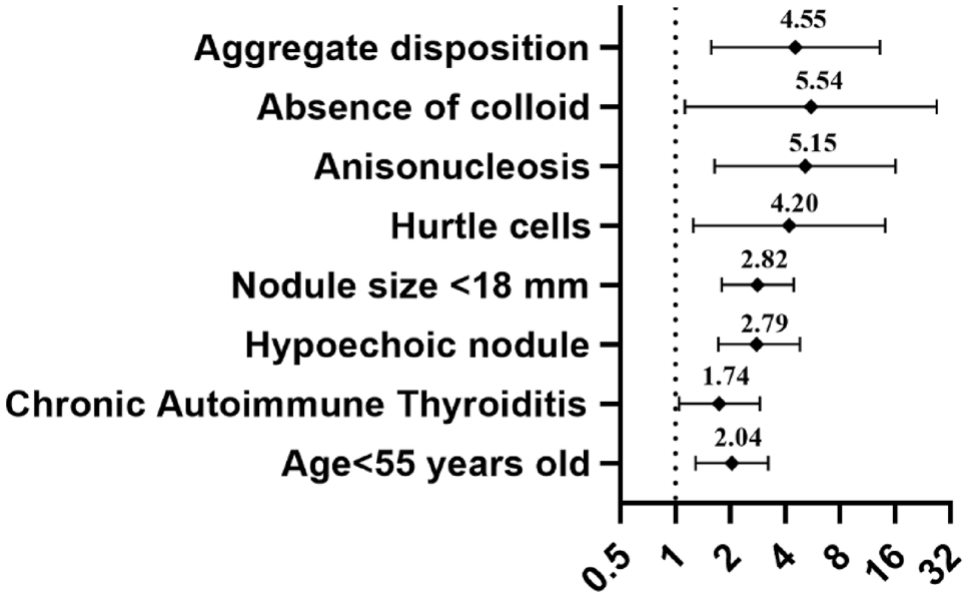
Forest plot of the clinical, ultrasound and cytological features of the predictive algorithm and their magnitude (odds ratio) in predicting the positive histology

A unified malignancy-predicting algorithm has been built based on the aforementioned multivariate analysis. For uniformity and graphical purposes, the favourable predictors have been transformed into their opposite (i.e., unfavourable, e.g., “absence of”) to build a homogeneous positive score for thyroid malignancy (see Fig. 1). The final algorithm represents the positive summation of the weight of each risk factor (i.e., each odds ratio), as derived from Table 4. In particular, the total score of each cytology derives from the sum of each feature value (if present).

**Fig. 2:**
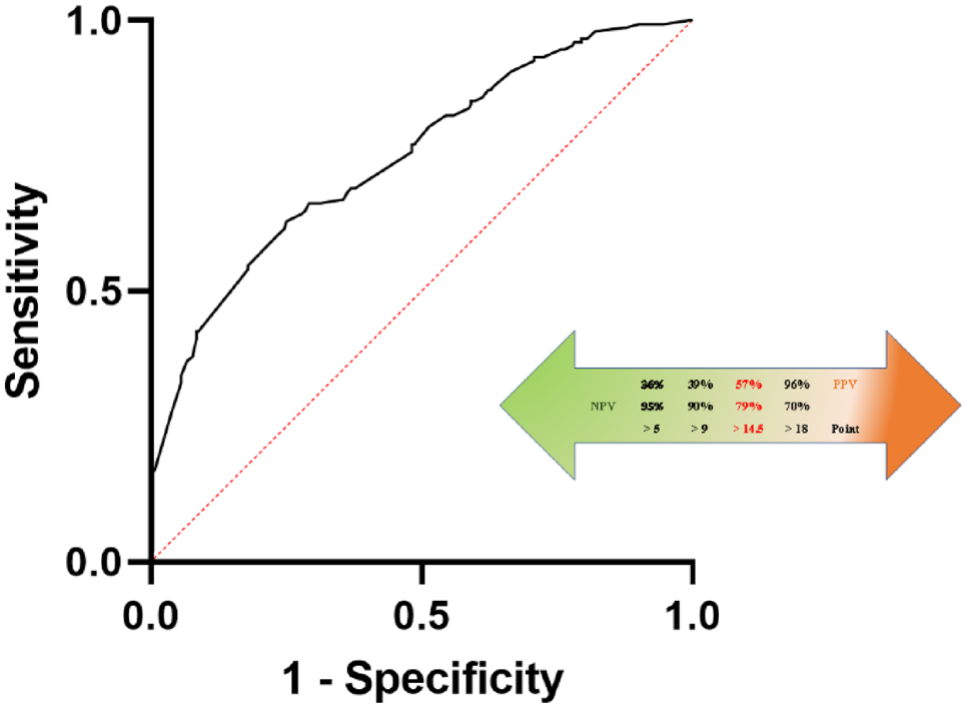
ROC curve analysis of the final algorithm (Phase 1), with graphic representation of the negative and positive predictive values of the main scale-points

To verify the prediction value of the new algorithm, we performed a ROC curve analysis considering the histological outcome as a readout. As shown in Fig. 2, the algorithm predicts an unfavourable outcome with high accuracy (AUC = 0.748, 95% CI 0.699–0.797, *p* < 0.0001). For a score > 14.5, we obtained a sensitivity of 60.1% and a specificity of 76.8% in predicting the unfavourable outcome, which corresponds to an OR = 4.98 (95% CI 3.24–7.65, *p* < 0.0001). Similarly, having a total score > 14.5 corresponds to PPV = 57.4% and NPV = 78.7% (Fig. 2).

### Sensitivity analysis

To explore the individual predictive ability of either clinical or cytological features among all the significant features in multivariate analysis, two separate sub-algorithms were calculated. We calculated separately the clinical and cytological OR from Table 4 to build a clinical algorithm and a cytological algorithm. The clinical information included the cut-off age, the presence of CAT, and the nodule features (i.e., cut-off size and hypoechogenicity). ROC curve analysis shows an accuracy of 0.715 (95% CI 0.663–767, *p* < 0.001). At value > 4.8 points, the sensitivity was 64.2% and the specificity was 68.3%, with a corresponding OR = 3.6 (95% CI 2.33–5.52, *p* < 0.0001). Considering only the cytological features (i.e., absence of colloid, HC, anisonucleosis and aggregate disposition), the accuracy of the ROC analysis was 0.635 (95CI 0.582–0.687, *p* < 0.001). At value > 9.5 the sensitivity was 68.3% and the specificity was 52.4%, with a corresponding OR = 2.44 (95% CI 1.63–3.67, *p* < 0.0001).

### Confirmatory analysis (phase 2)

To explore the predictive ability of the aforementioned algorithm, a new and independent, but smaller, population (*N* = 58, 15.3% of all cytology) has been tested. Table 2 shows the histological results, while Table 5 summarises the main features of the confirmatory sample. Interestingly, exploratory and confirmatory populations were mostly comparable. In fact, no differences were found when comparing the rates of positive and negative histology (*p* = 0.382) as well as in the majority of the features included in the algorithm (not shown). Only a few significant differences were observed. Considering clinical features, the confirmatory sample showed a higher prevalence of female patients (87.9%, *p* = 0.045) and a lower rate of hypoechoic nodules (39.7%, *p* < 0.001). Considering the cytological features, a lower rate of follicular configuration (46.6%, *p* = 0.003) and aggregate disposition (51.7%, *p* = 0.002) and the regular presence of macrophages (100%, *p* < 0.001) were found in the Phase 2 cohort.

By applying the algorithm from the exploratory sample to the confirmatory one, ROC curve analysis indicates a significant accuracy in predicting malignancy (AUC = 0.67, 95% CI 0.58–0.832, *p* = 0.043), even in this small sample. Patients who had a total score > 14.5 showed a comparable higher risk of positive histology with an OR = 4.64 (95% CI 1.36–15.82, *p* = 0.014) vs. OR = 4.98 of the exploratory analysis. When applied to the confirmatory sample, the threshold > 14.5 shows a PPV and NPV of 52.9% and 80.5%, respectively.

## Discussion

The present study shows that combining the often available clinical, US, and cytological information can improve the oncological stratification of a significant proportion of TIR3B cytology. This approach can support clinicians in the surgical selection of suspected nodules, without additional costs for both patients and public health systems. Of note, the present algorithm shows that a global score > 14.5 points improves the commitment toward a surgical indication almost twice (PPV = 57% and NPV = 79%), compared to the expected malignancy based on guidelines (< 30%) [1]. Similarly, scoring less than 14.5 reduces by up to 5% the risk of false negative results, downgrading the expected oncological risk of TIR3B cytology to that of TIR3A ones. As a consequence, if confirmed in other studies, in the event of very low scoring, some TIR3B nodules can be clinically followed up without the immediate need for surgical intervention.

The added value of the present study is that it exclusively focuses on TIR3B cytology, all of which is referred for surgery. It is, therefore, a homogeneous patient’ sample. In addition, results were verified in a smaller, but comparable, population, consistent with a clinical daily life.

Since the new Italian SIAPEC–IAP classification has been issued, an overall increase in the indeterminate cytology rates has been observed [16, 25]. The proportion of both TIR3A and TIR3B results ranges from 14% to 24% of all the cytology [3, 16]. Of those, almost half of the cases are represented by TIR3B, corresponding to as many surgical candidates. Even if growing evidence is in favour of a higher rate of thyroid cancer within the TIR3B category [16, 17], such as to justify the surgical resolution, this indication still represents an overtreatment in the majority of cases. This therapeutic attitude is tantamount to that of similar indeterminate categories in comparable international classifications, such sFN of the Bethesda classification [2] or the “neoplasm possible/suggesting follicular neoplasm-Thy3f” of the British one [26]. Likewise, the aforementioned categories share the same prediction limitation.

Regarding the outcome prediction, less progress has been achieved, and most of the oncological stratification efforts proved to be a clinical defeat. For instance, despite the great interest in molecular analyses, their applications to indeterminate cytology are difficult to evaluate on a large scale. Some studies show the potential advantages of large molecular panels, which include the major genes involved in thyroid cancer development [13, 14]. The most popular tests are represented by the Afirma Genomic Sequencing Classifier (Afirma GSC), ThyGeNEXT/ThyraMIR (MPTX), and Thyroseqv3 (TSv3), which analyse DNA, RNA or both. All the panels disclose high NPV, but larger prospective validations are pending and their use is very uneven, especially outside the United States [27]. These gaps, along with the high cost of the molecular analyses, make them the prerogative of only a few institutes, representing a limited diagnostic fringe in the indeterminate cytology field. Small PCR panels based on the most frequent thyroid molecular targets (i.e., *BRAF-V600E*, *N-H-K-RAS*, and *RET/PTC 1-3* fusions) are currently available in most Institutions, including ours. These panels are more cost-effective, but usually reserved for TIR3A nodules, since in TIR3B cytology the risk of positive histology remains high despite molecular results, as shown in a limited patients cohort [28].

The present study’s purpose is to maximise the commonly available information facing TIR3B nodules by improving the oncological stratification. To do this, we decided to merge the most significant clinical and US features with the cytological ones. In fact, the availability of detailed cytology reports allowed a uniform and reliable analysis of secondary cytological characteristics, unveiling potential additional predictors in this category. Very few studies have analysed the TIR3B population from this perspective. In addition, the present cohort is also the largest so far studied in this context. Finally, to the best of our knowledge, this is the only study that has simultaneously weighted the risk of a comprehensive panel of secondary cytological features, including the controversial HC. In fact, for TIR3B, the morphological appearance of the thyrocytes is well-structured with increased cellularity and discrete cellular patterns. All this information is available in the cytological reports released by our institution [18].

Cozzolino et al. [29] performed a similar analysis, but on a small sample of 96 TIR3B nodules. Despite the significant differences in the population-size, we observed some similar predictors, i.e., the cut-off age of 55 years and a comparable nodule-size threshold (20 vs. 18 mm). However, the Authors’ model did not include any cytological features and has not been verified in a confirmatory sample [29]. Another study based on the Bethesda cytological classification [30] considered 233 cytology, but only 44 sFN—comparable to our TIR3B. A lower cut-off age (45 years) and some US features (microcalcifications, irregular borders and solitary nodules) were found as independent predictors, at the multivariate analysis [30]. However, this study compared several categories, and no specific cytological details have been provided [30].

Considering HC, different evidences from the literature should be pointed out. These cytological types usually fall under indeterminate cytology and lead to interpretation challenges, because they can be found in both benign and malign histology. To date, conflicting data have emerged, and the occurrence of HC has also been related to the aging process and the presence of thyroiditis [31]. It is well-known that chronic inflammation may determine cellular changes, including the appearance of numerous mitochondria, resulting in the typical HC oncocytic phenotype. Thus, due to the high prevalence of CAT, it has often been found in these contexts. However, in a sample of 345 indeterminate cytology, classified partially according to the Bethesda [2] and then to the SIAPEC–IAP classification [1], the coexistence of HC and CAT has been associated with a lower rate of positive histology (6.2% vs. 32%, *p* = 0.005) [31]. In that study [31], no other factors were explored. In addition, Perticone results are in contrast with those of Pu et al. [32] who, in a dated but focused study on HC prognostic role, found no difference in cancer rates according to HC cytology. In the present study, despite the presence of 26% of CAT, only 8% of subjects showed concurrent HC positivity, with no differences in the histological results. Furthermore, even if at univariate analysis the HC presence seemed to support a favourable outcome after correcting for all the variables, including CAT, the role of HC resulted as an unfavourable, independent predictor. Another study [33] evaluated the role of HC on a smaller sample of 69 indeterminate cytology according to the Bethesda classification, including 62 sFN. Interestingly, the authors built a multivariate model of cytological predictors with some analogies to ours, consisting of “absence of colloid” (OR = 13.38, *p* = 0.002), “size > 2.9 cm” (OR = 8.55, *p* = 0.002), “non-uniform HC population” (OR = 4.01, *p* = 0.044) and “cellularity high” (OR = 6.65, *p* = 0.011). However, several criticisms should be highlighted. While the nodule size and the absence of colloid are variables that could be easily determined, the quantification of cellularity and the uniformity of HC introduce two operator-dependent items, adding further difficulties to the cytological descriptions among different pathologists. Moreover, at variance with the Yuan study [33], we found that larger nodules are associated with a lower oncological risk. This finding confirms previous reports showing that the large indeterminate nodules (i.e., > 30 mm) did not harbour a higher risk of malignancy [34]. From another perspective, it is worth noting that the follicular variant of papillary thyroid carcinoma, one of the most frequent thyroid cancer subtypes, is often diagnosed within a previous indeterminate cytology, especially in the event of small nodules, then revealing thyroid carcinoma (i.e., microcarcinoma) [12].

The present results show that an overall assessment of nodules underlying a TIR3B cytology is effective in better estimating the global oncological risks of a significant proportion of this indeterminate cytology. In fact, the merging of clinical and cytological risk factors leads to an additive effect, since each individual perspective carries a specific prognostic hazard, as shown in the sensitivity analysis.

Finally, the strength of these results is further supported by their reliability in a smaller confirmatory cohort sample, where the outcome is consistent with that of the stratification algorithm. This point has a double implication: on one hand, it endorses the validity of the exploratory analysis. On the other hand, it proves that the same algorithm can be effectively applied to small patient’ samples or single cases, which represent the daily occurrence in a clinical setting.

Although the retrospective design was a forced choice for the specific study purpose, we must recognize that the monocentric data represents a limitation of the present results. In particular, albeit already observed in other studies [16–19], we know that a selection bias could affect the higher rate of thyroid cancer diagnosed in our tertiary centre cohort. Furthermore, even if the current algorithm does not fully overcome the need for diagnostic surgery, it might significantly support physicians in better estimating patients’ oncological risks. The largest cohort sample and the uniformity of the clinical, cytological, and histological information, strongly endorse the present findings. In this light, upon the confirmation of this algorithm’s validity in other prospective and multicentre cohorts, the proposed clinical and cytological algorithm will shed light on a more tailored definition of the indeterminate category.

### Supplementary Information

Below is the link to the electronic supplementary material.

Supplementary file1 (DOCX 18 KB)

## Data Availability

Restrictions apply to the availability of some or all data generated or analysed during this study to preserve patient confidentiality or because they were used under license. The corresponding author will on request detail the restrictions and any conditions under which access to some data may be provided.
