# Supplementary material for: Improving the TIR3B oncological stratification: try to bridge the gap through a comprehensive presurgical algorithm
Source: J Endocrinol Invest. 2023 Sep 22;47(3):633–43. doi: 10.1007/s40618-023-02182-5 (PMC10904402; doi:10.1007/s40618-023-02182-5)
Supplement: Supplementary file 1 — Supplementary file1 (DOCX 18 KB) [file 40618_2023_2182_MOESM1_ESM.docx]

| Supplementary Table 1. Nodule ultrasound features. | |
| --- | --- |
|  | Description |
| Position | Superior/medium/inferior;  Anterior/posterior, paraisthmic/isthmic;  Distance from the thyroid capsule (sub-capsular) |
| Size | Report the three diameters for each nodule:   - depth or anteroposterior - width or transversal - length or longitudinal |
| Composition | - Solid: composed almost entirely of solid tissue with < 10% liquid - Mixed predominantly solid: liquid component > 10% but < 50% - Mixed predominantly cystic: liquid component > 50% but < 90% - Cystic: composed entirely or nearly entirely of liquid (> 90%) - Spongiform appearance: tiny cystic spaces separated by thin septa |
| Shape | - Round: equal length of the three diameters - Oval: depth less than width and depth less than length - Irregular: neither oval nor round, including ‘taller than wide’ (depth > width) and ‘taller than long’ (depth > length) shape |
| Echogenicity | - Isoechoic: brightness similar to the surrounding thyroid parenchyma. When the echogenicity of the surrounding thyroid tissue is decreased, such as in Hashimoto’s thyroiditis, the echogenicity can be compared to that of the sub-mandibular salivary glands - Hyperechoic: brighter appearance in comparison with the surrounding thyroid parenchyma - Mildly hypoechoic: darker appearance in comparison with the surrounding thyroid parenchyma, but less dark than neck strap muscles - Markedly hypoechoic: dark appearance, similar or higher than that of the neck strap muscles |
| Margins and halo | - Ill-defined margins: lack of clear demarcation with respect to the surrounding thyroid parenchyma. Ill-defined margins are distinct from irregular margins and do not alter the nodule risk category - Irregular margins: the presence of one or more sharp angles of the margins (spiculated) or the presence of one or more smooth focal round protrusions of the margins (lobulated) - Halo/rim: describe as thin or thick, partial or complete |
| Echogenic foci and calcifications | - Microcalcifications: punctate echogenic foci, ~ 1 mm, within the solid component of the nodule; - Macrocalcifications: hyperechoic foci > 1 mm with posterior shadowing; - Indeterminate hyperechoic foci: punctate hyperechoic foci that could not be definitively classified as microcalcifications, without posterior shadowing or artifacts; - Hyperechoic foci with comet-tail artifact, within nodule cystic component. |
| Vascularization | - Type I: absence of intranodular or perinodular flow - Type II: presence of perinodular and/or slight intranodular flow - Type III: presence of marked intranodular flow; mixed pattern (i.e., peri- and intranodular marked vascular flow) |
| Adapted from Rago T, et al. doi: 10.1007/s40618-018-0935-8 and Grani G, et al. doi: 10.1210/clinem/dgad092 | |
